# Spontaneous skull base cerebrospinal fluid leak during pregnancy: a case report and review of the literature

**DOI:** 10.1186/s12884-023-05460-5

**Published:** 2023-03-08

**Authors:** Lauren Michelle, Rebecca J. Post, Edward C. Kuan, Michael P. Nageotte

**Affiliations:** 1 Department of Otolaryngology – Head and Neck Surgery, University of California Irvine Medical Center, Orange, CA USA; 2 Department of Obstetrics and Gynecology, University of California Irvine Medical Center, 3800 West Chapman Ave, Ste 3800, Orange, CA USA; 3 Division of Maternal-Fetal Medicine, Miller Children’s and Women’s Hospital, Long Beach, CA USA

**Keywords:** Cerebrospinal fluid leak, Pregnancy, Neuraxial anesthesia, Rhinorrhea, Idiopathic intracranial hypertension, Case report

## Abstract

**Background:**

Idiopathic intracranial hypertension can lead to dural defects and spontaneous leakage of cerebrospinal fluid (CSF) from the skull base. Skull base CSF leaks are rarely reported in pregnancy but pose unique challenges for obstetricians and anesthesiologists.

**Case presentation:**

A 31-year-old G4P1021 at 14 weeks developed debilitating headaches and CSF rhinorrhea. Brain imaging revealed a bony defect of the sphenoid sinus with a meningoencephalocele and a partially empty sella, consistent with CSF leakage from a skull base defect. The patient was neurologically stable without signs of meningitis; thus, management was focused on symptomatic alleviation. A planned cesarean section was performed at 38 weeks under spinal anesthesia. The patient had spontaneous marked improvement of her symptoms postpartum.

**Conclusion:**

Pregnancy may exacerbate skull base CSF leaks, requiring careful management with a multidisciplinary team. Neuraxial anesthesia can safely be performed in pregnant individuals with spontaneous skull base CSF leakage, but further studies are needed to determine the safest mode of delivery in these patients.

## Teaching points

1. Individuals with a spontaneous skull base cerebrospinal fluid (CSF) leak may experience worsening of symptoms during pregnancy.
2. Neuraxial anesthesia can safely be performed in pregnant individuals with spontaneous skull base CSF leakage and is preferred over general anesthesia when possible.
3. Mode of delivery in pregnant individuals with skull base CSF leakage should be individualized with involvement of a multidisciplinary team and patient shared decision-making.

## Background

Cerebrospinal fluid (CSF) leaks from the skull base most often results from head and/or maxillofacial trauma, iatrogenically through surgery, congenitally through encephaloceles, or as anticipated from otolaryngologic and/or neurosurgical procedures, but can also occur spontaneously in some cases without any known precipitating event. These spontaneous CSF leaks most likely occur secondary to idiopathic intracranial hypertension (IIH), for which high body-mass index and female sex are correlated risk factors [1–3]. The increased intracranial pressure (ICP) from IIH could lead to development of dural defects in the anterior (typically from the sphenoid sinus, frontal sinus, or cribriform plate) or lateral (most commonly tegmen tympani) skull base and result in leakage of CSF through the nasal passages or into the middle ear [1, 4]. Appropriate management of skull base CSF leaks is imperative due to the potentially life-threatening complications that can arise, including meningitis and herniation of brain structures [1, 5]. Case reports describing spontaneous skull base CSF leak during pregnancy are exceedingly rare, thus posing a challenge for obstetricians and anesthesiologists seeking to determine the safest mode of delivery and method of anesthesia to avoid these potential complications. Using terms “(spontaneous cerebrospinal fluid leak OR CSF rhinorrhea) AND pregnancy”, three case reports (*n* = 3) were found prior to 2022 (published between 1979 and 2012) involving spontaneous CSF leak secondary to skull base defect in pregnancy were found via PubMed search and citation review [4, 6, 7]. The case described here discusses the unique presentation and management of a symptomatic pregnant patient with skull base CSF leak.

## Case presentation

**Fig. 1:**
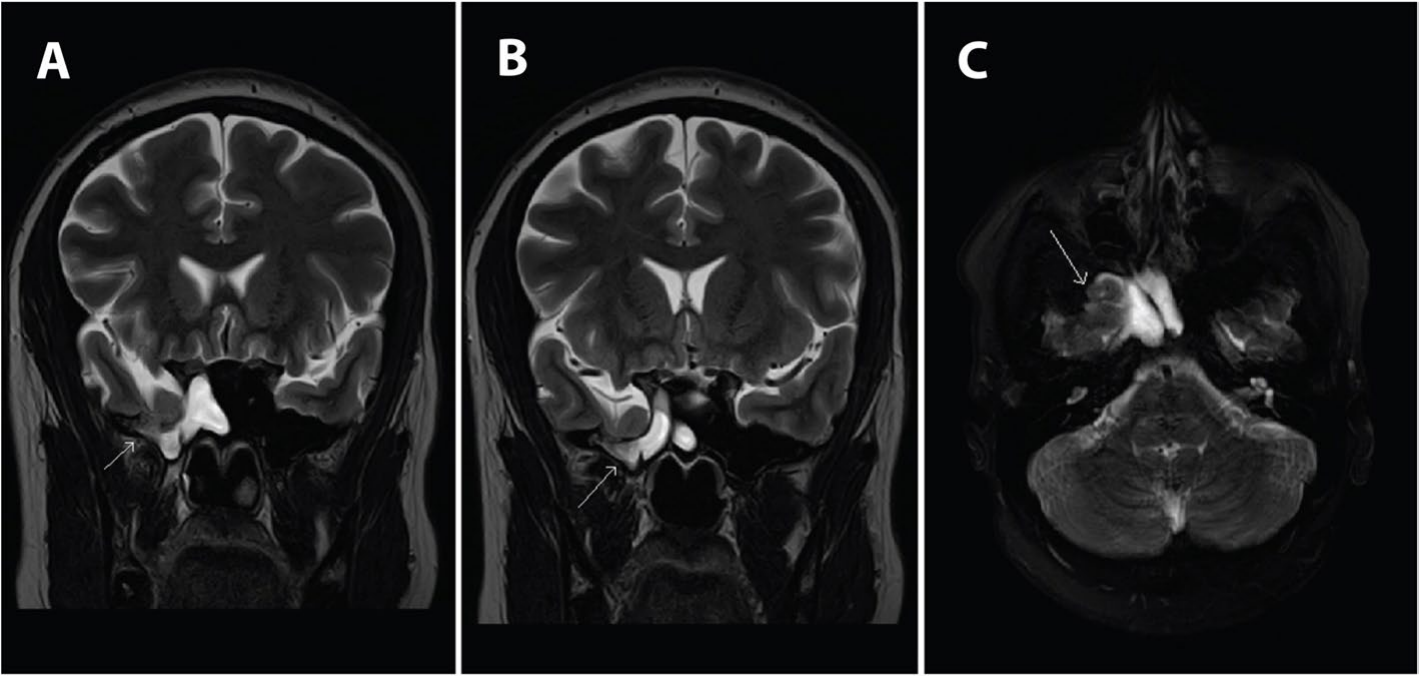
**A** & **B** Adjacent slices of a coronal T2-weight MRI showing a focal defect in the floor of the middle cranial fossa and a meningoencephalocele continuous with defect. **C** T2 axial view of the same findings

A 31-year-old G4P1021 at 14 weeks gestation was referred by her primary obstetrician to otolaryngology for new onset debilitating headaches and persistent right-sided clear fluid rhinorrhea for nearly 4 months, slightly pre-dating conception of her pregnancy. Her nasal fluid tested positive for beta-2 transferrin, consistent with CSF leak [8]. She was evaluated by neurosurgery, who obtained brain magnetic resonance imaging (MRI) and computed tomography (CT) scan without contrast. Imaging showed an osseus defect along the floor of the middle cranial fossa/roof of the right sphenoid sinus with a meningoencephalocele involving the right temporal lobe which extended through the bony defect (Fig. 1). A partially empty sella was noted, and an air fluid level was visualized in the right sphenoid sinus suggestive of CSF. The patient was otherwise healthy, with a pregnancy complicated only by maternal obesity (BMI 38). and microcytic anemia requiring iron infusions. She had no other medical problems, history of trauma, or previous surgical history with otolaryngology or neurosurgery. The otolaryngology and neurosurgery consultants recommended postponing surgical intervention until after delivery. Management of her pregnancy was subsequently transferred to a Maternal Fetal Medicine specialist at 30 weeks gestation with plan to deliver at a tertiary care medical center. The patient’s headaches were treated with hydration, acetaminophen, caffeine, and butalbital-acetaminophen-caffeine with codeine as needed. Daily nausea was managed with ondansetron as needed. Due to the frequency and severity of her headaches and rhinorrhea, the patient was unable to work throughout her pregnancy and was placed on disability. After a multidisciplinary discussion with neurosurgery, anesthesiology, and Maternal Fetal Medicine specialists, and shared decision making with the patient, the decision was made to proceed with scheduled cesarean delivery at 38 weeks under spinal anesthesia to avoid Valsalva during labor. The patient underwent a planned, uncomplicated primary low transverse cesarean section, and delivered a viable female infant weighing 2850 g, with Apgar scores of 8 and 9 at 1 and 5 min, respectively. Postpartum course was unremarkable, and patient reported significant improvement in the frequency and severity of her headaches after delivery, which was managed with ibuprofen. At last follow-up 8 months postpartum, the patient was neurologically stable, without symptoms of meningitis, and had planned on neurosurgical repair of her skull base defect; however, surgery was canceled due to improvement in her headaches and the patient’s desire to avoid a hospital setting during the COVID-19 pandemic.

## Discussion and conclusions

Spontaneous skull base CSF leak is a rare event (accounting for an estimated 2% of all CSF leaks) [1] and even more uncommonly reported in pregnant women, with just three cases published in the literature to date. While skull base CSF leakage occurs most commonly after head trauma or neurosurgery, spontaneous leakage is thought to be secondary to IIH [1–3]. Despite this presumed etiology, patients may present without any typical symptoms of high intracranial pressure (ICP), potentially due to regular siphoning of CSF acting as a natural shunt [2]. Management of CSF rhinorrhea includes conservative medical treatment with head-of-bed elevation ≥ 30 degrees, use of stool softeners, and avoidance of Valsalva maneuvers; however, surgical repair is regarded as a first-line therapy for patients experiencing chronic symptoms [1]. Serious but rare complications of untreated skull base CSF leak include meningitis or CSF hypovolemia resulting in headache, pneumocephalus, or brainstem herniation [1]. In published cases of pregnant women with skull base CSF leak, patients experienced symptom onset either in the first or third trimester, and management typically required surgical repair for definitive treatment postpartum or during pregnancy in one case [4, 7].

Table 1 summarizes the reported cases of skull base CSF leakage occurring in pregnancy, including our patient’s case. The first reported case of skull base CSF leak in a pregnant woman described a 21-year-old G1 at 35 weeks gestation who developed left-sided nasal CSF rhinorrhea after the pushing portion of a Lamaze exercise, but was otherwise asymptomatic. She notably had a history of pneumococcal meningitis twice with CSF rhinorrhea at age 17. She later presented after spontaneous rupture of fetal membranes, received epidural anesthesia, and her labor was augmented with oxytocin, and she delivered 3 h later via forceps-assisted vaginal delivery. Her CSF rhinorrhea initially resolved after delivery, but subsequently recurred two weeks postpartum [6]. The second case involved a 34-year-old G2P0 who developed daily CSF rhinorrhea during her first trimester, 11 months after undergoing endoscopic surgery for chronic sinusitis. She had no headache or other symptoms. She was delivered via elective cesarean delivery with spinal anesthesia. Her rhinorrhea persisted postpartum, and she underwent unsuccessful endoscopic repair at 3 months postpartum with recurrent CSF leakage [7]. The third case concerned a 34-year-old woman presenting with right-sided hearing loss and clear fluid leaking from the nose. Beta-2 transferrin testing was positive, and she was incidentally found to be at 13 weeks gestation on routine pregnancy test. Computed tomography (CT) scan showed bilateral osseous defects in the ethmoid roof with CSF leakage. The patient underwent surgical repair of the defects during her pregnancy, and subsequently delivered a healthy male infant 6 months later via elective caesarean section (method of anesthesia was not specified) with no reported recurrence of symptoms on 2 year follow-up [4]. In contrast to the previously reported cases, our patient case is unique in that she experienced debilitating headaches in addition to rhinorrhea, both of which significantly improved postpartum. Due to our patient’s chronic CSF rhinorrhea, stable neurologic state, and lack of vision problems, management was focused on symptomatic alleviation. Thus, our patient was treated conservatively during her pregnancy with bedrest and analgesic medication to manage her symptoms with the plan for surgical repair postpartum.

**Table 1:** Summary of reported cases of skull base cerebrospinal fluid leakage in pregnancy

Case	Relevant History	Symptoms	Onset	Treatment	Delivery	Anesthesia	Postpartum
Weinstein et al. 1979 [6]	History of meningitis with CSF rhinorrhea s/p repair 4 years prior	CSF rhinorrhea	3^rd^ trimester	Expectant management	Forceps-assisted vaginal delivery	Epidural	Recurrence of CSF rhinorrhea 2 weeks postpartum, planned surgical repair
Schabel et al. 2002 [7]	Prior endoscopic surgery 11 months prior	CSF rhinorrhea	1^st^ trimester	Expectant management	Elective primary cesarean section	Spinal	Persistent CSF rhinorrhea after delivery, underwent surgical repair at 3 months that was unsuccessful
Schraven et al. 2012 [4]	None	CSF rhinorrhea and ear leakage	1^st^ trimester	Surgical repair during pregnancy	Elective primary cesarean section	Not specified	Asymptomatic at 2-year follow-up
Michelle et al. 2023 (this study)	None	CSF rhinorrhea and severe headaches	1^st^ trimester	Analgesics and bed rest	Elective primary cesarean section	Spinal	Marked improvement in symptoms after delivery, plan to have surgical repair

*CSF* Cerebrospinal fluid, *s/p *Status post

Our patient’s development of headaches during pregnancy and subsequent postpartum improvement is related to her skull base defect and possible IIH. One explanation is that while CSF pressure is generally unaltered (7-15mmHg) during the course of a normal pregnancy, physiologic changes as well as weight gain in pregnancy could increase intracranial pressures, possibly exacerbating her baseline risk factors of female sex and obesity [3, 9]. These changes likely predisposed her to develop symptomatic IIH and subsequent spontaneous skull base CSF leak, with partial alleviation of her headaches after delivery. While our patient did not receive a lumbar puncture to confirm a diagnosis of IIH, her imaging findings of empty sella and meningoencephalocele are classic imaging signs of elevated ICP [2]. Additionally, a lumbar puncture may not have reflected IIH given the patient’s chronic nasal leakage of CSF.

Though not utilized in our patient’s case, acetazolamide which is commonly employed in the treatment of IIH has also been suggested as a potential treatment for spontaneous CSF skull base leaks in a small retrospective study by Tilak and colleagues in 2019. Acetazolamide was administered pre-operatively to 16 non-pregnant patients with spontaneous CSF rhinorrhea and found that 31.3% of the patients had resolution of their symptoms without the need for surgical repair, though it was most effective when combined with weight loss [10]. Acetazolamide has traditionally been avoided in pregnant women due to teratogenic effects in animal studies, but has been prescribed in pregnancy for IIH without apparent adverse fetal effects in small clinical studies, and thus could be considered as an alternative treatment option when conservative measures fail [11, 12].

Given the rarity of skull base CSF leakage reported in pregnancy, the safest mode of delivery and choice of anesthesia for a patient with skull base CSF leakage is not clear. The first and second stage of labor are known to increase CSF pressure, which could potentially worsen CSF leakage and lead to complications such as brain herniation. Similarly, general anesthesia can result in significant increases of intracranial pressure, especially during airway manipulation or Valsalva during intubation and extubation. However, creating dural defects during administration of spinal anesthesia or inadvertently during epidural anesthesia also has the possible risk of causing additional CSF leakage, though this risk is small, particularly with spinal anesthetic using a small gauge needle [9, 13]. Several case reports included in this review reported uncomplicated use of both epidural and spinal anesthesia in vaginal and cesarean deliveries in patients with spontaneous skull base CSF leaks [4, 6, 7]. Considering our patient had significant neurological symptoms (unlike the other reported cases of skull base CSF leaks in pregnancy) and weighing the risks of these procedures, our patient decided to proceed with cesarean delivery under spinal anesthesia to avoid possible exacerbation of her skull base defect. Without high-quality evidence-based guidance, mode of delivery and anesthesia should be individualized with involvement of a multidisciplinary team and patient shared decision-making.

Pregnant patients presenting with similar symptoms to our patient should be evaluated for IIH and/or skull base spontaneous CSF leak. Women with skull base CSF leaks may have worsening of their symptoms during pregnancy, but can be managed conservatively with intravenous fluids and/or analgesic medication, or if severe or intractable, with surgery. Though successful use of regional anesthesia and forceps-assisted vaginal delivery has been reported in patients with skull base CSF leaks, very little is definitively known about risks during pregnancy, and thus further studies are needed to determine the safest mode of delivery and anesthesia.

## Data Availability

Not applicable as this is a case report.

## Abbreviations

CSF: Cerebrospinal fluid
CT: Computed tomography
ICP: intracranial pressure
IIH: Idiopathic intracranial hypertension
MRI: Magnetic resonance imaging
